# Editorialets

**Published:** 1910-09

**Authors:** 


### Editorialets.

---

ROOSEVELT'S MAXIMS.—Fit yourself for the work God has for you to do in this world and lose no time about it.

---

Have all the fun that is coming to you.

---

Go ahead, do something and be willing to take responsibility.

---

Learn by your mistakes.

---

A REMARKABLE FEAT.—"Dr. Abraham Jacobi recently averred that he took a cold plunge 365 times a year and was sure he should have died twenty times of pneumonia or some other 'providential' disease without it. However, he considers a cold plunge too severe for the anemic, the very young or the old."—*Southern Medical Journal, Chattanooga.*

Most men only die once. But Jacobi is a remarkable man and could die twenty times and not feel it, I suppose.

---

DR. BRUMBY will resign the office of State Health Officer of Texas November 1, to become medical director of an insurance company at Houston.

---

PRESERVE HOGG'S LAST SPEECH.—Dr. M. M. Carrick, physician at the State Epileptic Colony at Abilene, has purchased the record containing the last speech of the late Governor James S. Hogg, and he will present it to Hon. O. B. Colquitt, Democratic nominee for Governor, to be presented to the State for preservation in the Department of Archives and History. When Dallas entertained the members of the Twenty-ninth Legislature, Governor Hogg was ill at Fort Worth. He had been invited to address the members of the Legislature in this city, but, being physically unable to do so, he used the phonograph for a brief speech, which was repeated by phonograph at the dinner. It is these remarks, the last public utterances of the Governor, which the record in question preserves.—*From Dallas News,* July 29, 1910.

